# MicroRNA-210 induces apoptosis in colorectal cancer via induction of reactive oxygen

**DOI:** 10.1186/s12935-016-0321-6

**Published:** 2016-06-10

**Authors:** Katrin E. Tagscherer, Anne Fassl, Tabea Sinkovic, Jutta Richter, Sabrina Schecher, Stephan Macher-Goeppinger, Wilfried Roth

**Affiliations:** Molecular Tumor-Pathology, German Cancer Research Center (DKFZ), 69120 Heidelberg, Germany; Institute of Pathology, University of Heidelberg, 69120 Heidelberg, Germany; Institute of Pathology, University Medical Center Mainz, Langenbeckstraße 1, 55131 Mainz, Germany; Department of Cancer Biology, Dana-Farber Cancer Institute, Boston, MA 02215 USA; Department of Genetics, Harvard Medical School, Boston, MA 02215 USA

**Keywords:** miR-210, Colorectal carcinoma, ROS, Bim, Apoptosis

## Abstract

**Background:**

Deregulation of miRNA-210 is a common event in several types of cancer. However, increased expression levels in the cancer tissue have been associated with both poor and good prognosis of patients. Similarly, the function of miR-210 with regard to cell growth and apoptosis is still controversial.

**Methods:**

Overexpression of miR-210 was performed in HCT116, SW480 and SW707 colorectal cancer (CRC) cell lines. Functional effects of a modulated miR-210 expression were analyzed with regard to proliferation, clonogenicity, cell cycle distribution, reactive oxygen species (ROS) generation, and apoptosis. Furthermore, quantitative real time (RT)-PCR and immunoblot analyses were performed to investigate signaling pathways affected by miR-210.

**Results:**

We show that in CRC cells miR-210 inhibits clonogenicity and proliferation which was accompanied by an accumulation of cells in the G2/M phase of the cell cycle. Additionally, overexpression of miR-210 results in an increase of ROS generation. Moreover, miR-210 mediated the induction of apoptosis which was associated with an upregulation of pro-apoptotic Bim expression and enhanced processing of Caspase 2. Importantly, inhibition of ROS generation rescued cells from miR-210-induced apoptosis.

**Conclusions:**

Taken together, miR-210 induces apoptosis in CRC cells via a ROS-dependent mechanism.

**Electronic supplementary material:**

The online version of this article (doi:10.1186/s12935-016-0321-6) contains supplementary material, which is available to authorized users.

## Background

Colorectal carcinoma (CRC) is the third most common cancer and one of the leading causes of death due to cancer world-wide [1]. Defects in the apoptosis signaling cascade account for resistance to therapy of malignant tumors. In the case of CRC this resistance to radio- and chemotherapy substantially contributes to a poor prognosis. So far, the molecular mechanisms underlying the varying degree of cell death resistance of CRC are largely unknown. Therefore, a better understanding of the regulation of survival and therapy resistance of CRC cells is urgently required.

MicroRNAs (miRNAs) are short (20–23 nucleotides) non-coding mRNA molecules [2] functioning as post-transcriptional regulators of gene expression. Since miRNAs contribute to the regulation of different cellular processes involving apoptosis, cell cycle regulation and differentiation, their deregulation quite often results in tumorigenesis [3]. MiRNAs are transcribed as so-called pri-miRNAs, which are cleaved by Drosha in the nucleus [4]. The resulting microRNA precursor molecules (pre-miRNAs) are subsequently transported into the cytoplasm and processed to the mature miRNA by the Dicer complex [4]. The guide strand is integrated into the RISC complex resulting in the degradation of target mRNAs or their transcriptional inhibition [4]. MiRNAs were first discovered in 1993 by Lee et al. [5]. Since then, approx. 1400 human miRNAs have been discovered, amongst them almost 400 to be deregulated in CRC [6].

So far, it has been shown that miR-210 is upregulated in a variety of human cancers, including lung cancer [7–9], renal cell carcinoma [10–13], pancreatic carcinoma [14], breast cancer [15], hepatocellular carcinoma [16], colorectal carcinoma [17, 18] and adrenocortical carcinoma [19, 20]. Besides, miR-210 is downregulated in squamous cell carcinoma [21] and ovarian cell carcinoma [22]. Although miR-210 overexpression is accompanied by a poor prognosis in many human tumors [15, 23–28], it has recently been shown, that high expression levels of miR-210 were significantly associated with an improved disease free survival in non-small cell lung cancer [29] and clear cell renal cell carcinoma post nephrectomy [30]. Similarly, low miR-210 expression levels were accompanied with a higher rate of relapse and a poorer treatment outcome in pediatric acute lymphoblastic leukemia [31]. Further, the function of miR-210 regarding the regulation of cell growth and apoptosis is quite controversial. Whereas some studies show that downregulation of miR-210 reduces viability in renal cell carcinoma [10], endothelial cells [32] and hepatoma [33], other studies claim that miR-210 acts in a pro-apoptotic manner in neuroblastoma [34], lung adenocarcinoma [35], renal cell carcinoma [36], esophageal squamous carcinoma [21] and lung adenocarcinoma [37].

In this study, we sought to explore the functional role of miR-210 with regard to apoptosis in CRC. We demonstrate that an increased expression of miR-210 reduces proliferation, cell cycle progression and colony formation. Furthermore, overexpression of miR-210 induces ROS generation and apoptosis, accompanied by an increased Bim expression and Caspase 2 processing. Taken together our results identify miR-210 as a potent inducer of apoptotic cell death in CRC and suggest the miR-210-induced ROS generation to be a possible key player within this process.

## Results

### miR-210 overexpression influences CRC growth

**Fig. 1:**
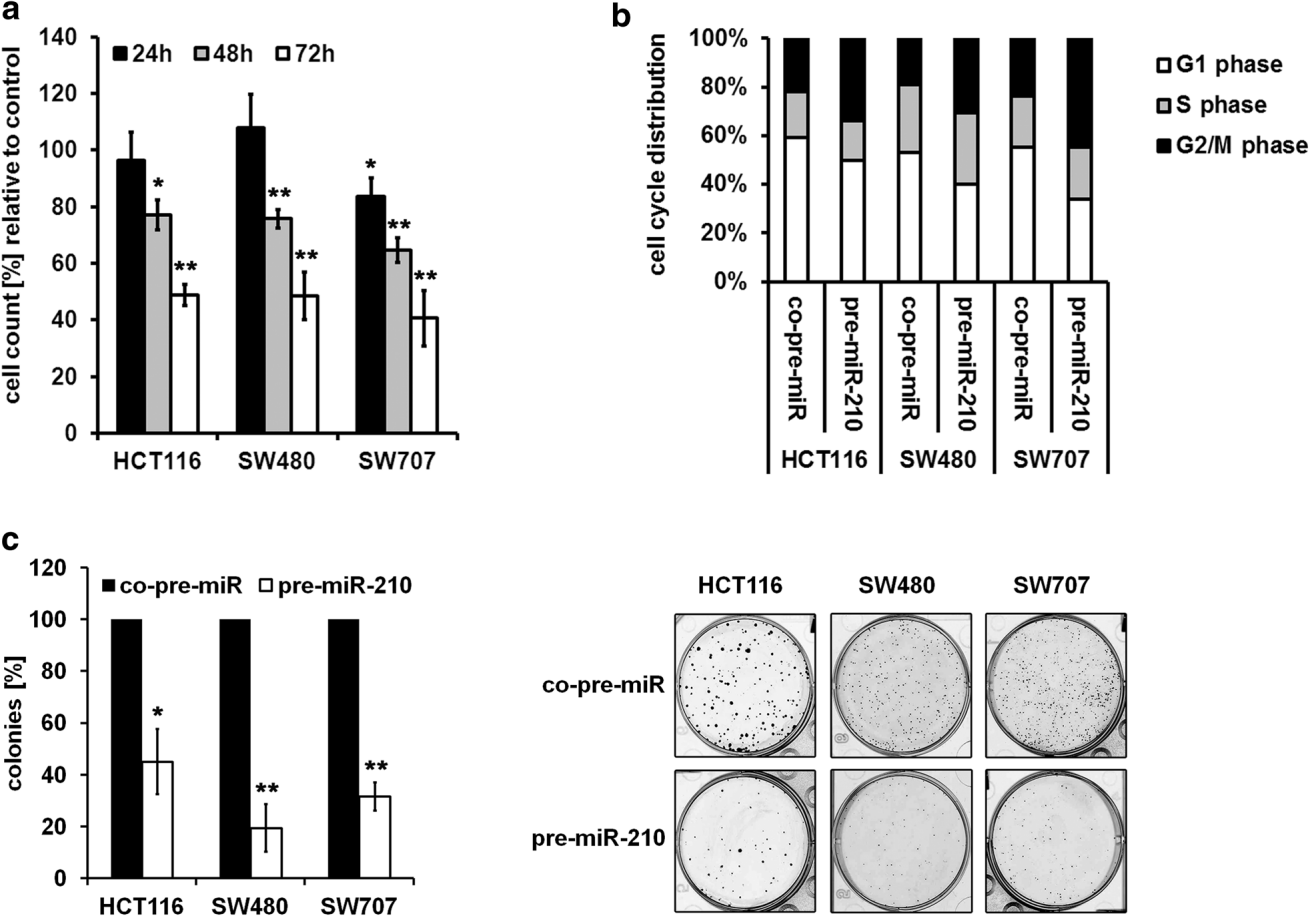
Overexpression of miR-210 inhibits cell growth. **a** Proliferation assay of CRC cell lines 24, 48 and 72 h after transfection with pre-miR-210 and a control miRNA, respectively. Cells were trypsinized and counted (mean ± SEM; n = 3; *p < 0.05; **p < 0.01; student’s t test). **b** Measurement of cell cycle distribution was performed 48 h after transfection of pre-miR-210 and a control miRNA, respectively. Cells were stained with PI and the DNA content was measured by FACS analysis. Data represent the mean of three independent experiments. **c** Pre-miR-210 and control miRNA transfected CRC cells were seeded into 6-well-plates (500 cells/well) 72 h post transfection and cultured for 7 days. The colonies were subsequently stained with *crystal violet* and counted (*left panel*; mean ± SEM; n = 3; *p < 0.05; **p < 0.01; student’s t-test). A representative picture is shown (*right panel*)

The role of miR-210 in the regulation of cell growth and death is quite controversial. Whereas some studies show that downregulation of miR-210 reduces viability in renal cell carcinoma [10], endothelial cells [32] and hepatoma [33], other studies claim miR-210 to act in a pro-apoptotic manner [21, 34–37]. To characterize the functional relevance of miR-210 in CRC we analyzed the effects of an increased expression of miR-210 on cell growth. To this end, we transfected colorectal cancer cell lines with a miR-210 precursor oligonucleotide (pre-miR-210; Additional file 1: Figure S1). Overexpression of exogenous miR-210 resulted in a significant reduction of proliferation (Fig. 1a) which was accompanied by a decrease of cells in the G1 phase and an accumulation of cells in the G2 phase of the cell cycle (Fig. 1b). In line with these results, miR-210 overexpression reduced clonogenicity (Fig. 1c).

### miR-210 overexpression induces apoptosis

**Fig. 2:**
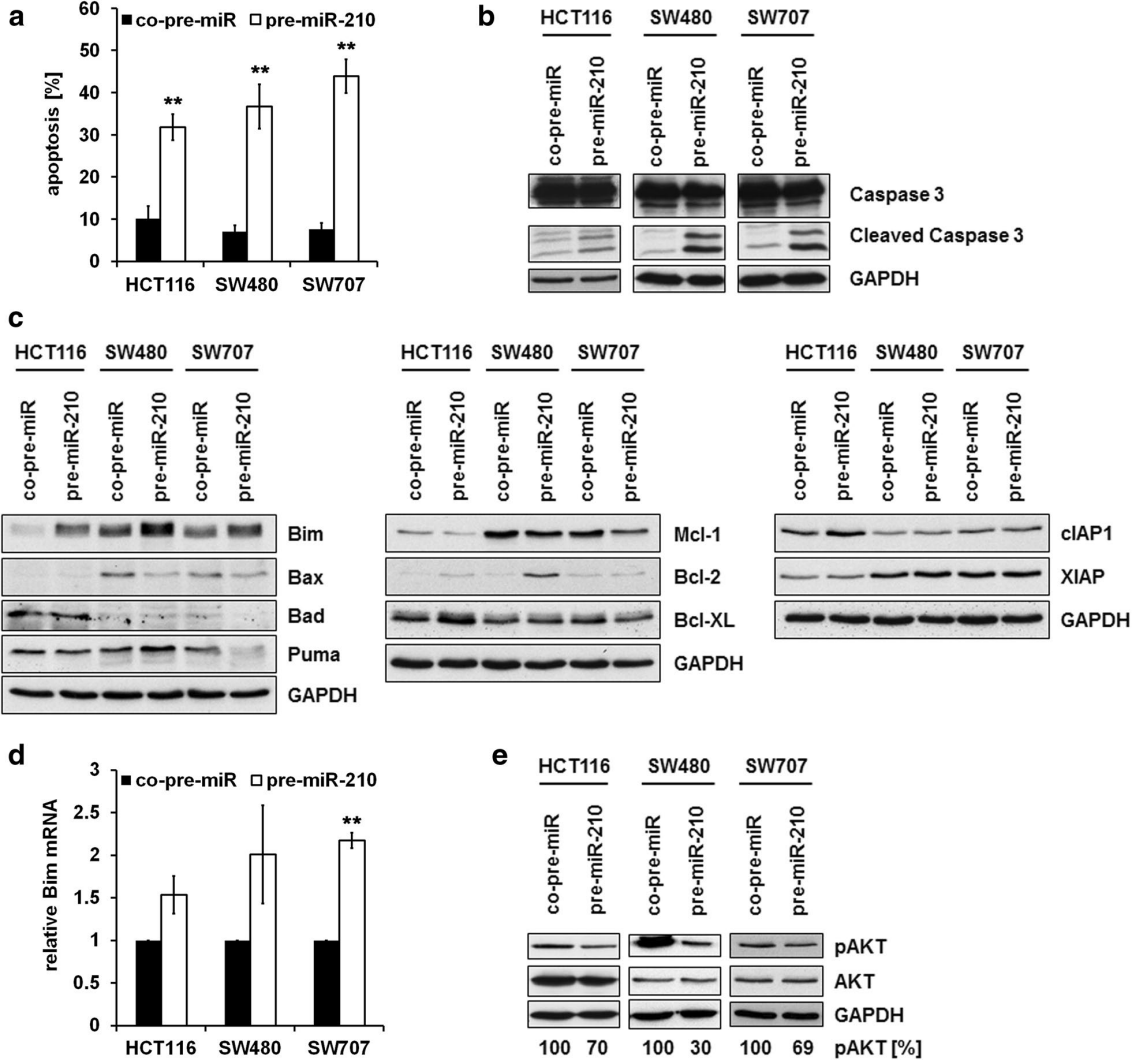
Overexpression of miR-210 induces apoptosis in CRC cell lines. **a** 72 h post transfection with pre-miR-210 and a control miRNA, respectively, the percentage of apoptotic cells was determined by PI staining and FACS analysis. Data indicate the percentage of cells showing a sub-G_1_-DNA content (mean ± SEM; n = 3; **p < 0.01; student’s t-test). Representative flow cytometric histograms are shown in Additional file 2: Figure S2. **b** CRC cell lines were transfected as described in A. Lysates were generated 72 h thereafter. Cleavage of Caspase 3 was determined by immunoblot analysis. **c** CRC cell lines were transfected as described in A. Lysates were generated 48 h thereafter. **d** qRT-PCR analysis of Bim mRNA expression in pre-miR-210 transfected CRC cell lines compared to control miRNA transfected cells. CRC cell lines were transfected as described in A. Isolation of RNA was carried out 48 h thereafter. The level of Bim mRNA expression was measured by quantitative RT-PCR analysis and normalized to internal 18S rRNA expression (mean ± SEM; n = 3; **p < 0.01; student’s t-test). **e** CRC cell lines were transfected as described in A. Lysates were generated 48 h (HCT116, SW707) and 72 h (SW480) thereafter. pAKT expression levels were determined by immunoblot analysis. pAKT expression levels were densitometrically quantified using ImageJ Software and normalized to total AKT expression

To further functionally analyze the effects of increased miR-210 expression levels, we investigated the impact of miR-210 on cell death. Overexpression of miR-210 potently induced apoptotic cell death (Fig. 2a; Additional file 2: Figure S2), which was accompanied by cleavage of Caspase 3 (Fig. 2b). To characterize miR-210 mediated apoptosis, we analyzed the expression levels of different pro- and anti-apoptotic proteins. Immunoblot analyses revealed a consistent downregulation of the anti-apoptotic Mcl-1 protein and an upregulation of the pro-apoptotic Bim protein upon miR-210 overexpression in all cell lines examined. The expression levels of other pro- or anti-apoptotic proteins which have additionally been analyzed, were not or only marginally affected (Fig. 2c). MiR-210 mediated upregulation of Bim involves transcriptional mechanisms as demonstrated by quantitative PCR analyses of Bim mRNA (Fig. 2d). Since the AKT pathway is known to regulate Bim expression on the transcriptional level via FOXO transcription factors [38], we were interested whether this pathway is likewise affected by miR-210. As a matter of fact, immunoblot analysis revealed that the AKT pathway is less activated in cells overexpressing miR-210 compared to control cells (Fig. 2e), pointing to an involvement of AKT in the regulation of Bim.

**Fig. 3:**
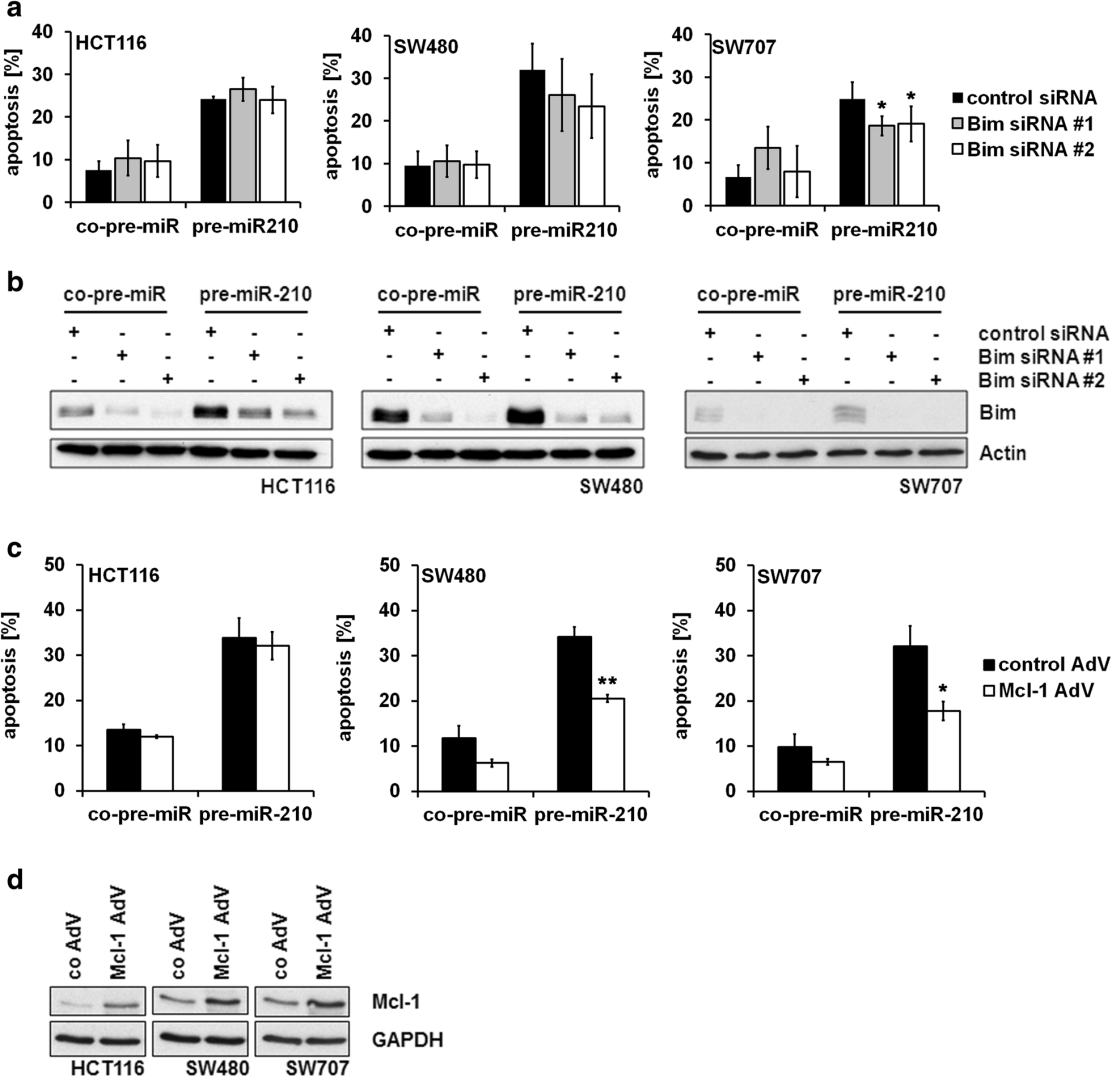
Downregulation of Bim and upregulation of Mcl-1 inhibit miR-210-mediated apoptosis. **a** CRC cell lines were transfected with pre-miR-210 and a control miRNA, respectively. Concomitantly, they were transfected either with two different Bim-specific siRNAs or a control siRNA. 72 h post transfection, the percentage of apoptotic cells was determined by PI staining and FACS analysis. Data indicate the percentage of cells showing a sub-G_1_-DNA content (mean ± SEM; n = 3; *p < 0.05; student’s t-test). **b** CRC cells were transfected as described in A. Lysates were generated 72 h thereafter. The successful knockdown of Bim was monitored by immunoblot analysis. **c** CRC cell lines were transduced with Mcl-1 AdV and a control AdV, respectively. 24 h thereafter, cells were transfected with pre-miR-210 or a control miRNA. The percentage of apoptotic cells was determined 72 h after transfection by PI staining and FACS analysis. Data indicate the percentage of cells showing a sub-G_1_-DNA content (mean ± SEM; n = 3; *p < 0.05; **p < 0.01; student’s t-test). **d** CRC cell lines were transduced with Mcl-1 AdV and a control AdV, respectively. Lysates were generated 72 h thereafter

Further, we investigated the impact of Bim and Mcl-1 on miR-210 mediated apoptosis. To this end, we simultaneously transfected miR-210 oligonucleotides and different siRNAs targeting Bim mRNA, which should counteract the pro-apoptotic effect of miR-210. Surprisingly, knockdown of Bim did not (HCT116) or only marginally (SW480 and SW707) inhibit miR-210 induced apoptosis (Fig. 3a, b). With regard to Mcl-1, CRC cell lines were transduced with an Mcl-1 AdV and transfected with miR-210 oligonucleotides. Overexpression of Mcl-1 significantly reduced miR-210 mediated apoptosis in SW480 and SW707 cells, however no effect on apoptosis rate in HCT116 cells was observed (Fig. 3c, d).

### Inhibition of ROS partially protects cells from miR-210 induced cell death

**Fig. 4:**
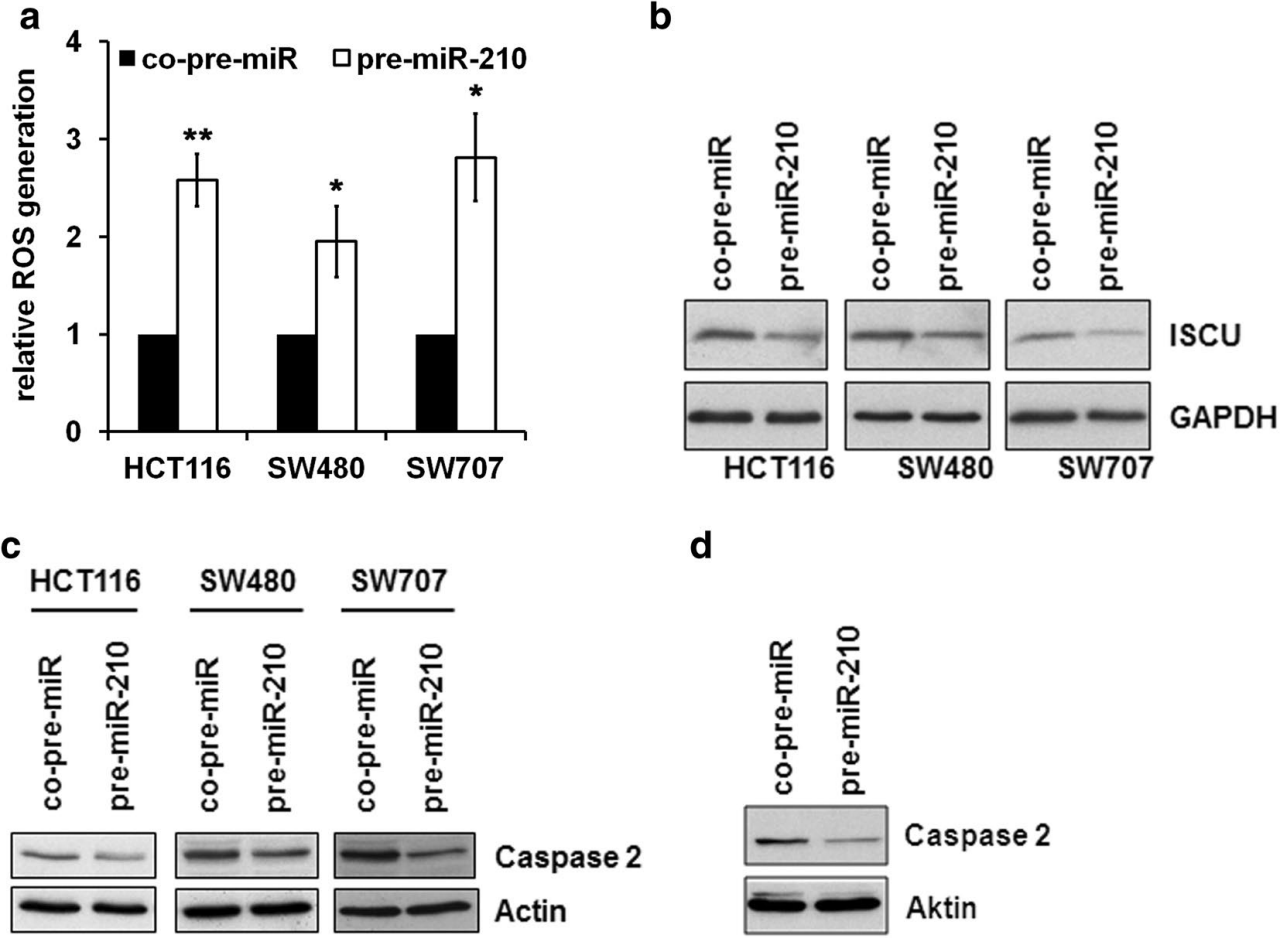
Overexpression of miR-210 induces ROS generation. **a** Measurement of ROS generation was performed 48 h after transfection of pre-miR-210 and a control miRNA, respectively (mean ± SEM; n = 3; *p < 0.05; **p < 0.01; student’s t-test). **b** CRC cell lines were transfected as described in A. Lysates were generated 48 h thereafter. ISCU expression was analyzed by immunoblot analysis. **c** CRC cell lines were transfected as described in A. Lysates were generated 48 h thereafter. Caspase 2 processing was analyzed by immunoblot analysis. **d** MCF-7 breast cancer cells were transfected as described in A. Lysates were generated 72 h thereafter. Caspase 2 processing was analyzed by immunoblot analysis

MiR-210 overexpression has been associated with an impairment of mitochondrial function in various cellular contexts [37, 39, 40]. These effects have mainly been associated with miR-210 mediated regulation of the Fe-S cluster scaffold protein ISCU [39–42]. Thus, we examined the effects of miR-210 overexpression on ROS generation and ISCU expression in our cellular system. Importantly, overexpression of miR-210 resulted in a substantial increase in ROS generation (Fig. 4a). Further, we observed a distinct decrease in ISCU expression upon transfection of miR-210 oligonucleotides (Fig. 4b). Moreover, siRNA mediated downregulation of ISCU increased cellular ROS generation and induced apoptosis (Additional file 3: Figure S3a, b). However, overexpression of ISCU did not counteract miR-210 mediated ROS and apoptosis induction (Additional file 3: Figure S3c, d). Since ROS is known to induce apoptosis via the activation of Caspase 2 [43, 44], we investigated its processing dependent on miR-210 expression. In fact, overexpression of miR-210 resulted in Caspase 2 cleavage (Fig. 4c). Additionally, as Caspase 3 is able to activate Caspase 2 [45, 46], we further wanted to exclude that the enhanced processing of Caspase 2 was exclusively mediated through a positive feed-back-loop resulting from an increased activation of Caspase 3. To this end, we transfected MCF-7 breast cancer cells, lacking Caspase 3 expression [47], with pre-miR-210 and control oligonucleotides, respectively. Immunoblot analysis revealed that overexpression of miR-210 provoked Caspase 2 processing in MCF-7 cells as well (Fig. 4d), thereby confirming that processing of Caspase 2 was independent of the activation of Caspase 3 within this context.

**Fig. 5:**
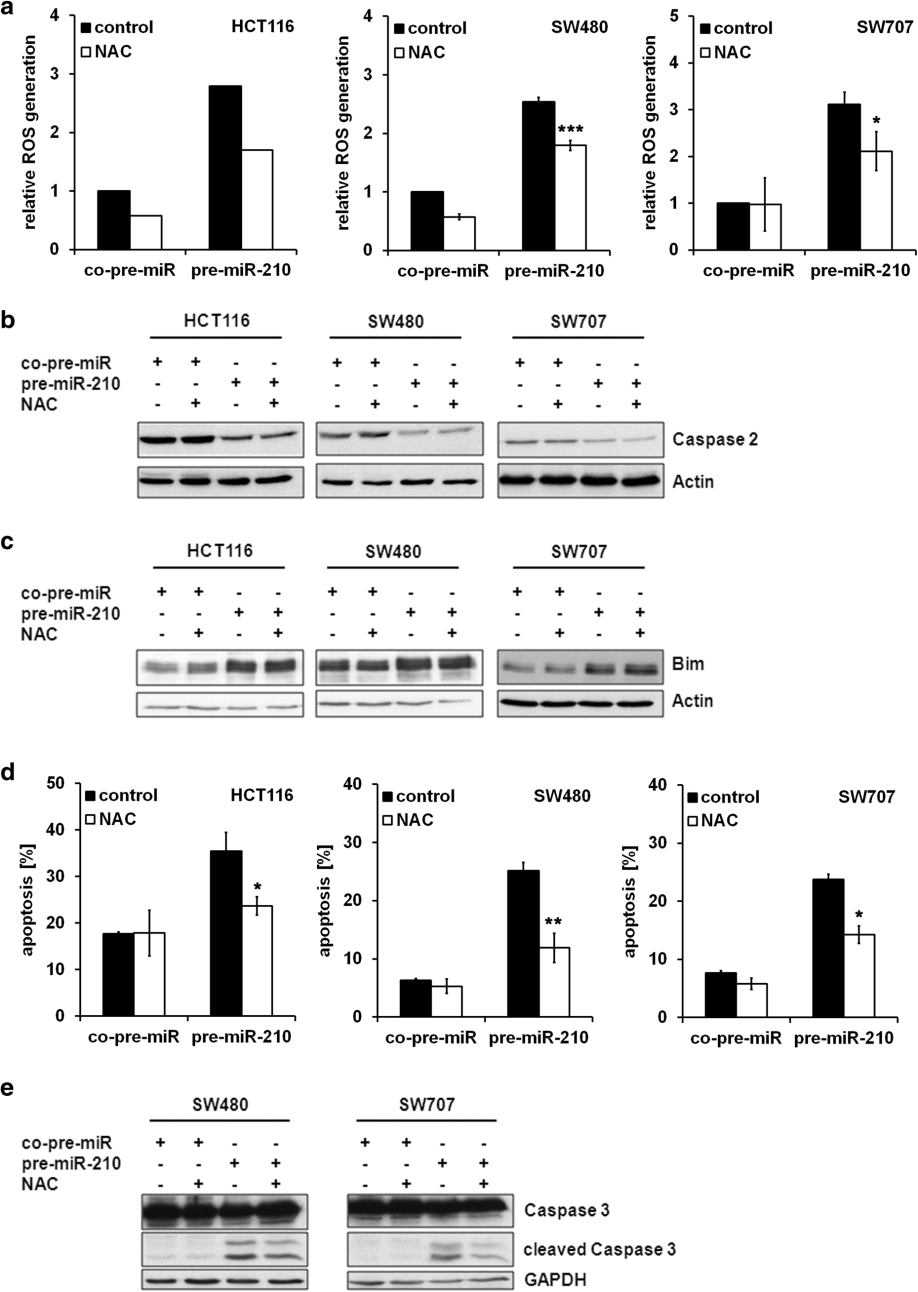
Inhibition of ROS generation affects miR-210 induced cell death. CRC cells were transfected with pre-miR-210 and a control miRNA, respectively. 24 h post transfection, cells were treated with NAC. **a** Measurement of ROS generation was performed 24 h post NAC treatment (30 mM) (HCT116: n = 1; SW480, SW707: n = 3; mean ± SEM; ***p < 0.001; *p < *0.05*; student’s t-test). **b** Lysates were generated 48 h post NAC treatment (10 mM). Processing of Caspase 2 was analyzed by immunoblot analysis. **c** Lysates were generated 48 h post NAC treatment (10 mM). Bim expression levels were determined by immunoblot analysis. **d** The percentage of apoptotic cells was determined 48 h post NAC treatment (30 mM) by PI staining and FACS analysis. Data indicate the percentage of cells showing a sub-G_1_-DNA content (mean ± SEM; n = 3; *p < 0.05; **p < 0.01; student’s t-test). **e** Lysates were generated 48 h post NAC treatment (10 mM). Cleavage of Caspase 3 was determined by immunoblot analysis

To further elucidate the role of ROS in miR-210 mediated apoptosis, cells were treated with the ROS scavenger N-acetylcysteine (NAC), which resulted in a decrease in miR-210 induced ROS generation (Fig. 5a). Although inhibition of ROS generation did neither affect expression levels of Bim, nor impair the cleavage of Caspase 2 (Fig. 5b, c), NAC treatment led to a considerable decrease in miR-210 mediated cell death (Fig. 5d) and to a diminished cleavage of Caspase 3 (Fig. 5e), thereby suggesting a role of ROS generation in miR-210 mediated cell death.

## Discussion

Altered expression of miR-210 can modulate either apoptosis resistance or sensitivity depending on the cellular context [10, 21, 32–37]. MiR-210 has been shown to be upregulated in CRC compared to normal tissue [17]. Further, high expression levels of miR-210 both in tumor tissue and serum of CRC patients correlate with a poor prognosis [17, 18]. Within this study we sought to explore the functional effects of an increased miR-210 expression in CRC.

Our results indicate that miR-210 functions in an anti-tumorigenic manner by decreasing proliferation accompanied by an increased amount of cells in the G2/M phase of the cell cycle. This is in accordance with previous studies showing an accumulation of cells in the G2 phase in various tumor entities upon miR-210 overexpression [35, 36, 48]. Moreover it has been shown that miR-210 overexpression induces senescence in fibroblasts [49] and reduces tumor growth and proliferation in hepatocellular xenografts [33]. Several direct and indirect targets of miR-210 might account for this effect. Zheng et al. previously demonstrated that overexpression of miR-210 blocks the expression of CyclinD1 and CyclinD2 via SHH/Gli1 signaling [50]. Additionally, the miR-210 target E2F3 [22, 36] plays an important role in regulation of proliferation [51]. He et al. further proposed Plk1, CyclinF, Bub1B, CDC25B and Fam83D to be involved in miR-210-mediated cell cycle arrest [48]. In addition to the direct targets of miR-210, proliferation arrest might be induced by an increased ROS generation. Several targets are known that are involved in cell cycle regulation and are regulated by ROS. The observed accumulation of cells in G2 phase might therefore be caused by the regulation of the oxidative state of Cdc25C. This phosphatase which activates cyclinB/cdk1 complexes, might be repressed by elevated ROS levels by inducing an inhibitory disulfide bond [52]. Besides a direct regulation of cellular proliferation, elevated amounts of ROS might contribute to the observed effects via activation of FOXOs. These transcription factors regulate the transactivation of a series of genes involved in cell cycle control [53]. Activation of the FOXO transcription factors might occur upon increased ROS levels by MST1, which is activated upon nuclear DNA damage [54] or by inhibition of the AKT kinase, which negatively regulates FOXOs [55]. Interestingly, we observed a decrease in phosphorylated AKT upon miR-210 overexpression. Similarly, Luo et al. recently demonstrated a ROS-dependent inactivation of AKT signaling accompanied with an increased activity of FOXO3a in colorectal cancer cells [56].

An increased generation of ROS upon miR-210 upregulation has so far been observed in colorectal carcinoma [42], in adipose-derived stem cells [57] and fibroblasts [49]. Furthermore, it has been shown that an elevated expression of miR-210 reduces oxygen consumption and upregulates glycolysis in various tumor entities [39, 40, 42, 58]. Within this context, it has been observed, that the activity of mitochondrial complex I [39, 40] and II [37] is impaired, resulting in an increased formation of ROS [54]. These effects have been proposed to be based on miR-210 mediated regulation of the Fe-S cluster scaffold protein ISCU [39–42], SDHD [37], a subunit of the succinate dehydrogenase complex, COX10 [42], a subunit of cytochrome c oxidase, and NDUFA4 [37], a subunit of the NADH dehydrogenase 1 alpha subcomplex. In line with these observations we could also detect a decreased expression of ISCU and NDUFA4 (data not shown) upon miR-210 overexpression. Whereas siRNA-mediated downregulation of ISCU increased ROS generation and induced apoptosis, siRNA-mediated downregulation of NDUFA4 did neither alter ROS generation nor apoptosis rates (data not shown). However, ectopic overexpression of ISCU did not counteract miR-210 mediated apoptosis and ROS generation. Therefore, it might by very likely, that ISCU is only one of several miR-210 targets regulating ROS generation. This is also evidenced by the effects of ISCU downregulation, since the reduced expression of ISCU did not completely reach the extent of miR-210 overexpression with regard to ROS generation and apoptosis induction.

Although there are several studies investigating the effect of a modulated miR-210 expression on apoptosis, the underlying molecular mechanisms are far from clear. Within this study we provide evidence, that an increased ROS generation induced by miR-210 overexpression contributes to the apoptotic phenotype. One of the most common pathways contributing to ROS-induced apoptosis is the ASK1 signaling cascade. Induction of ROS results in the oxidation of the inhibitory protein thioredoxin. Subsequently ASK1 and the downstream stress kinases JNK and p38 get activated, whereas the latter are able to induce cell death [59].

Besides the ASK1/JNK/p38 signaling axis, the transcription factor FOXO3 might contribute to the apoptotic effects upon ROS generation by transactivation of its target genes Bim, Bcl-6 and Noxa [54]. Within this context, FOXO3 might be activated by MST1 or inhibited by the AKT kinase [54, 55]. Accordingly, it was recently demonstrated that ROS-dependent inactivation of the AKT signaling pathway was accompanied by an increase in Bim expression levels [56]. Indeed, we detected an increase of Bim expression levels upon miR-210 overexpression, which was at least partially due to transcriptional regulation. Besides, ROS-induced ER stress and subsequent activation of the transcription factor CHOP might also contribute to the elevated Bim expression [38, 60]. However, siRNA-mediated downregulation of Bim did not (HCT116) or only slightly (SW480 and SW707) diminish miR-210-mediated apoptosis, pointing to a different mechanism triggering miR-210-mediated apoptosis. Furthermore, inhibition of ROS generation using NAC did not alter Bim expression levels, rendering a ROS-dependent regulation of Bim rather unlikely.

Interestingly, we observed a miR-210 mediated upregulation of the anti-apoptotic Bcl-2 protein in HCT116 and SW480 cells. So far it has been reported, that Bcl-2 overexpression can either inhibit or increase ROS induced apoptosis [61–63]. In this regard, we observed, that ectopic overexpression of Bcl-2 significantly reduced miR-210 mediated apoptosis (data not shown). Within this context it is tempting to speculate whether an increased expression of miR-210 sensitizes cancer cells to Bcl-2 inhibitors.

Within this study we could further demonstrate, that overexpression of miR-210 results in an increased processing of Caspase 2. Induction of ROS has been shown to induce activation of Caspase 2 [43, 44] in a both p53-dependent and –independent manner [64, 65] which might further result in apoptosis by Caspase 2-mediated cleavage of Bid or by directly inducing the release of Cyt c, AIF and SMAC from the mitochondria [66]. However inhibition of ROS generation did not alter Caspase 2 processing nor did Caspase 2 downregulation inhibit miR-210 induced cell death (data not shown).

The functional consequences of an increased miR-210 expression in cancer patients are so far unknown. It has been shown, that elevated miR-210 expression levels might either be beneficial [29–31] or be accompanied by a poor prognosis [15, 17, 18, 23–27]. However, it is still unclear whether the latter is directly caused by increased expression levels of miR-210. Since hypoxia is one of the main factors contributing to a poor prognosis of cancer patients [67], elevated miR-210 expression levels, which are mainly regulated by HIF transcription factors [25, 68, 69], might only be a bystander effect instead of directly influencing patients’ outcome. Therefore, the regulation of apoptosis by miR-210 might be of great biological relevance in CRC and warrants further investigation.

## Conclusions

Our experiments identify miR-210 as an inductor of apoptosis in CRC cells. Our results further identify miR-210-mediated increase in ROS generation as a key driver of miR-210-induced apoptosis.

## Methods

### Materials

N-acetylcysteine was obtained from Sigma-Aldrich (St. Louis, MO, USA, A9165). The antibodies were obtained as follows: anti-actin (Chemicon, Billerica, MA, USA, 1501); anti-AKT (Cell Signaling, Danvers, MA, USA, 9272); anti-Bad (Santa Cruz, Dallas, TX, USA, sc-7869); anti-Bax (Santa Cruz, sc-493); anti-Bcl-2 (Santa Cruz; sc-509); anti-Bcl-XL (Cell Signaling, 2764); anti-Bim (Cell Signaling, 2933); anti-Caspase 2 (Cell Signaling, 2224); anti-Caspase 3 (Imgenex, San Diego, CA, USA, IMG-144A); anti-cIAP1 (R&D Systems, Minneapolis, MN, USA, AF8181); anti-GAPDH (Santa Cruz, sc-32233); anti-ISCU (Santa Cruz; sc-373694); anti-Mcl-1 (Santa Cruz, sc-819); anti-pAKT (S472/473) (Cell Signaling, 4058); anti-Puma (Cell Signaling, 4976); anti-XIAP (Cell Signaling, 2045).

### Cell culture

The human colorectal cancer cell lines HCT116, SW480 and SW707 as well as the human breast cancer cell line MCF-7 were purchased from the American Type Culture Collection (ATCC, USA), maintained in RPMI medium (Life Technologies, Darmstadt, Germany) supplemented with 10 % fetal calf serum (Sigma-Aldrich), 1 mM glutamine, 25 mM glucose and 1 % penicillin/streptomycin (Life Technologies) and cultured at 37 °C in a 5 % CO_2_ atmosphere. Cell lines were regularly tested for the presence of contamination using multiplex cell contamination test [70] and authenticated by SNP profiling [71].

### Proliferation and clonogenicity assay

For the assessment of proliferation, cells were seeded into 6-cm culture dishes and counted after 24, 48 and 72 h using the trypan blue exclusion assay. For clonogenicity assays, 500 cells were seeded into 6–well culture dishes and incubated for 7 days prior to crystal violet staining and colony counting.

### FACS analysis

For analysis of cell cycle distribution and cell death, colorectal cancer cell lines were stained with propidium iodide (PI) as described previously [72].

For measurement of reactive oxygen species (ROS), colorectal cancer cells were seeded in 6-cm plates and transfected as indicated. Cells were incubated with the fluorescent H2DCF-DA (2,7-dichlorodihydrofluorescein diacetate; 5 µM; Biozol, Eching, Germany) for 30 min at 37 °C.

Cells were subjected to flow cytometry analysis using a Becton–Dickinson FACScalibur cytometer and Cell Quest Software.

### Transfections

Colorectal cancer cells were transiently transfected with siRNA using Lipofectamine 2000 (Life Technologies). Pre-miR-210 (miR precursor; PM10516) and co-pre-miR (control; AM17110) oligonucleotides were obtained from Life Technologies and used in a concentration of 50 nM. Bim siRNA #1 and #2 were obtained from Thermo Scientific (Waltham, MA, USA, #D-004383-18, #D-004383-17) and used in a concentration of 25 nM. ISCU siRNA was obtained from Life Technologies (#s23908) and used in a concentration of 5 nM. A non-specific siRNA served as a control (Thermo Scientific, #D-001810-01).

The pcDNA3-ISCU plasmid was generated by PCR from the clone pENTR221-ISCU, provided by the ORFeome Collaboration (Genomics and Proteomics Core Facility, DKFZ, Heidelberg, Germany) using the following forward (F) and reverse (R) primers containing *Bam*HI and *Xho*I restrictions sites: 5′- ATGCATGCATGGATCCACCATGGCGGCGGCTGGGGCT -3′ (F) and 5′- ATGCATGCATCTCGAGCAAGAAAGCTGGGTCCAATTTC -3′ (R). The PCR products were digested with *Bam*HI and *Xho*I and cloned into the correspondent sites of pcDNA3-Flag. For the generation of stable transfectants, complete medium containing Geneticin^®^ (G418, Invitrogen) at a concentration of 1.5 mg/mL was used to select stably transfected cells.

### Adenoviral transduction

Mcl-1-AdV was produced as described previously [73]. The control AdV consists of the empty AdV5 backbone and was kindly provided by Stefan Herzig (DKFZ, Heidelberg, Germany). CRC cells were incubated with recombinant AdVs directly after seeding using a multiplicity of infection of 10 (HCT116) or 200 (SW480, SW707).

### Immunoblot analysis

Cellular lysate generation and immunoblot analysis were performed as described previously [72]. Densitometric analyses were performed using ImageJ software (National Institutes of Health, Bethesda, MD, USA, http://www.rsb.info.nih.gov/ij/).

### Quantitative PCR analysis

Quantitative real-time PCR was performed as described previously [73]. Following primer pairs were used: Bim: 5′-CAACACAAACCCCAAGTCCT-3′ (forward), 5′-TCTTGGGCGATCCATATCTC-3′ (reverse); 18S: 5′-CATGGCCGTTCTTAGTTGGT-3′ (forward), 5′ ATGCCAGAGTCTCGTTCGTT-3′ (reverse).

For measurement of miRNA expression, total RNA was isolated using the miRNeasy Mini Kit (Qiagen, Hilden Germany). Mature miRNAs were reversely transcribed using TaqMan^®^ MicroRNA reverse transcription kit (Thermo Scientific) and TaqMan^®^ MicroRNA Arrays (Thermo Scientific). Quantitative PCR analysis was performed using the TaqMan^®^ Universal PCR Master Mix (Thermo Scientific) and a 7300 Real-Time PCR System (Applied Biosystems, Foster City, CA, USA). All steps were carried out according to the to the manufacturer’s protocols.

### Statistical methods

Significant differences were identified using the unpaired 2-sided Student *t* test. Throughout, *p* values <0.05 were considered significant and are indicated as follows: *p < 0.05, **p < 0.01, ***p < 0.001.

## Additional files

10.1186/s12935-016-0321-6 CRC cell lines were transfected with pre-miR-210 and a control miRNA, respectively. 48 h thereafter, total RNA including miRNA was isolated. Expression of miR-210 was measured by qRT-PCR analysis and normalized to internal U47 snoRNA expression (mean ± SEM; n = 3).
10.1186/s12935-016-0321-6 Representative flow cytometric histograms of CRC cell lines 72 h post transfection with pre-miR-210 and a control miRNA, respectively. Cells were stained with by PI staining and subjected to FACS analysis.
10.1186/s12935-016-0321-6 **a** CRC cell lines were transfected with an ISCU specific siRNA and a control siRNA, respectively. ROS generation was measured 48 h after transfection (left panel; mean ± SEM; n = 3; **p* < 0.05; ***p* < 0.01; student’s t-test). Successful knockdown of ISCU was monitored by immunoblot analysis. Lysates were generated 48 h after transfection (right panel). **b** Cells were transfected as described in a. The percentage of apoptotic cells was determined 72 h post transfection by PI staining and FACS analysis. Data indicate the percentage of cells showing a sub-G1-DNA content (mean ± SEM; n = 3; ***p* < 0.01; student’s t-test). **c** CRC cell lines stably overexpressing ISCU or an empty vector control (neo) were transfected with pre-miR-210 and a control miRNA, respectively. Measurement of ROS generation was performed 48 h thereafter (left panel; mean ± SEM; n = 3). Successful overexpression of ISCU-Flag was monitored by immunoblot analysis. **d** Cells were transfected as described in c. The percentage of apoptotic cells was determined by PI staining and FACS analysis (mean ± SEM; n = 3).

## Abbreviations

AdV: adenovirus
CRC: colorectal cancer
NAC: N-acetylcysteine
ROS: reactive oxygen species
