# Supplementary material for: MicroRNA-210 induces apoptosis in colorectal cancer via induction of reactive oxygen
Source: Cancer Cell Int. 2016 Jun 10;16:42. doi: 10.1186/s12935-016-0321-6 (PMC4901463; doi:10.1186/s12935-016-0321-6)
Supplement: Supplementary file 1 — 10.1186/s12935-016-0321-6 CRC cell lines were transfected with pre-miR-210 and a control miRNA, respectively. 48 h thereafter, total RNA including miRNA was isolated. Expression of miR-210 was measured by qRT-PCR analysis and normalized to internal U47 snoRNA expression (mean ± SEM; n = 3). [file 12935_2016_321_MOESM1_ESM.pdf]

Supplemental Figure S1

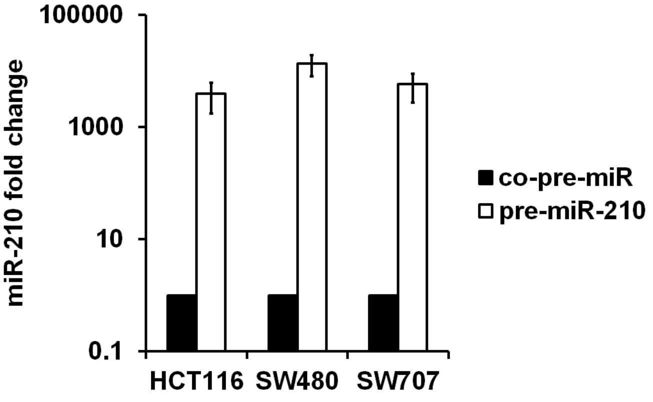

Supplemental figure S1. CRC cell lines were transfected with pre-miR-210 and a control miRNA, respectively. 48 h thereafter, total RNA including miRNA was isolated. Expression of miR-210 was measured by qRT-PCR analysis and normalized to internal U47 snoRNA expression (mean ± SEM; n=3).
