# Supplementary material for: MicroRNA-210 induces apoptosis in colorectal cancer via induction of reactive oxygen
Source: Cancer Cell Int. 2016 Jun 10;16:42. doi: 10.1186/s12935-016-0321-6 (PMC4901463; doi:10.1186/s12935-016-0321-6)
Supplement: Supplementary file 2 — 10.1186/s12935-016-0321-6 Representative flow cytometric histograms of CRC cell lines 72 h post transfection with pre-miR-210 and a control miRNA, respectively. Cells were stained with by PI staining and subjected to FACS analysis. [file 12935_2016_321_MOESM2_ESM.pdf]

Supplemental Figure S2

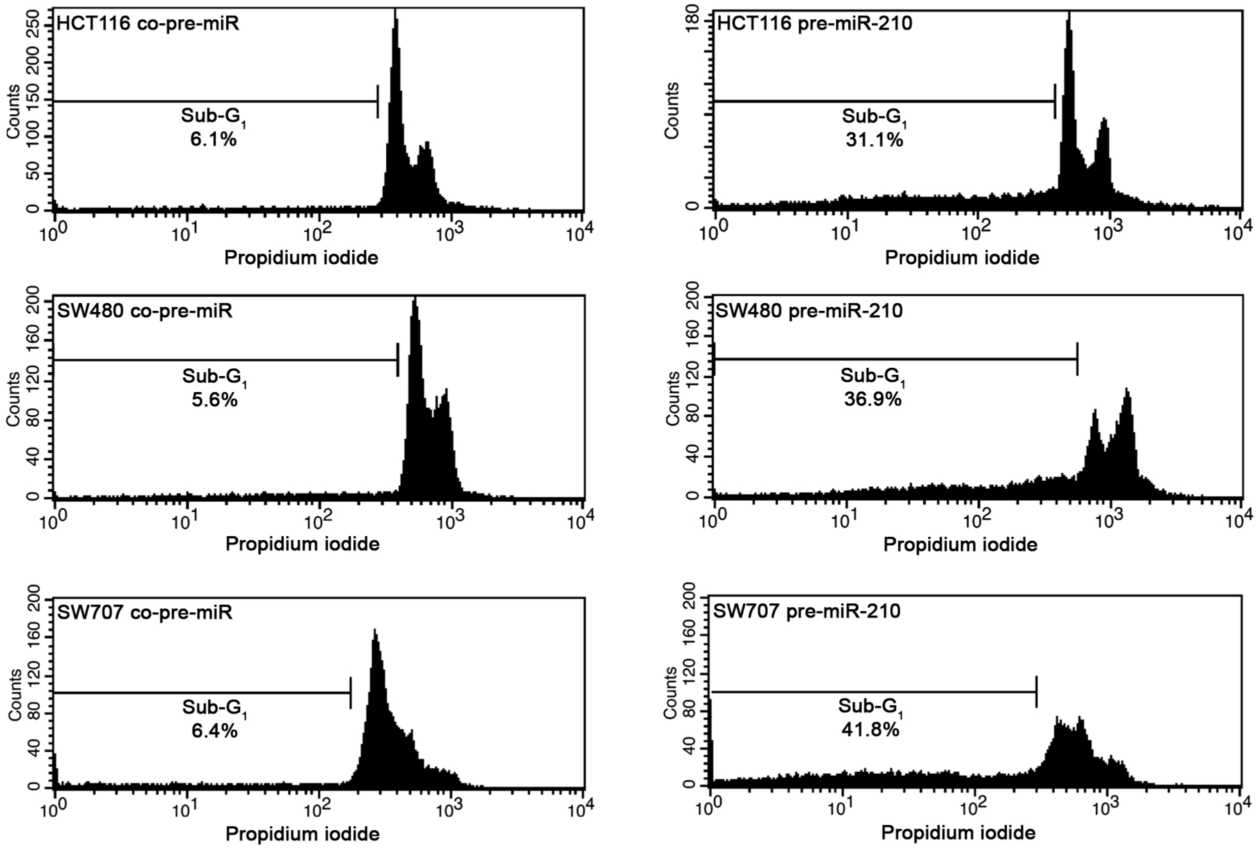

Supplemental Figure S2. Representative flow cytometric histograms of CRC cell lines 72 h post transfection with pre-miR-210 and a control miRNA, respectively. Cells were stained with by PI staining and subjected to FACS analysis.
