# Supplementary material for: MicroRNA-210 induces apoptosis in colorectal cancer via induction of reactive oxygen
Source: Cancer Cell Int. 2016 Jun 10;16:42. doi: 10.1186/s12935-016-0321-6 (PMC4901463; doi:10.1186/s12935-016-0321-6)
Supplement: Supplementary file 3 — 10.1186/s12935-016-0321-6 a CRC cell lines were transfected with an ISCU specific siRNA and a control siRNA, respectively. ROS generation was measured 48 h after transfection (left panel; mean ± SEM; n = 3; *p < 0.05; **p < 0.01; student’s t-test). Successful knockdown of ISCU was monitored by immunoblot analysis. Lysates were generated 48 h after transfection (right panel). b Cells were transfected as described in a. The percentage of apoptotic cells was determined 72 h post transfection by PI staining and FACS analysis. Data indicate the percentage of cells showing a sub-G1-DNA content (mean ± SEM; n = 3; **p < 0.01; student’s t-test). c CRC cell lines stably overexpressing ISCU or an empty vector control (neo) were transfected with pre-miR-210 and a control miRNA, respectively. Measurement of ROS generation was performed 48 h thereafter (left panel; mean ± SEM; n = 3). Successful overexpression of ISCU-Flag was monitored by immunoblot analysis. d Cells were transfected as described in c. The percentage of apoptotic cells was determined by PI staining and FACS analysis (mean ± SEM; n = 3). [file 12935_2016_321_MOESM3_ESM.pdf]

Supplemental Figure S3

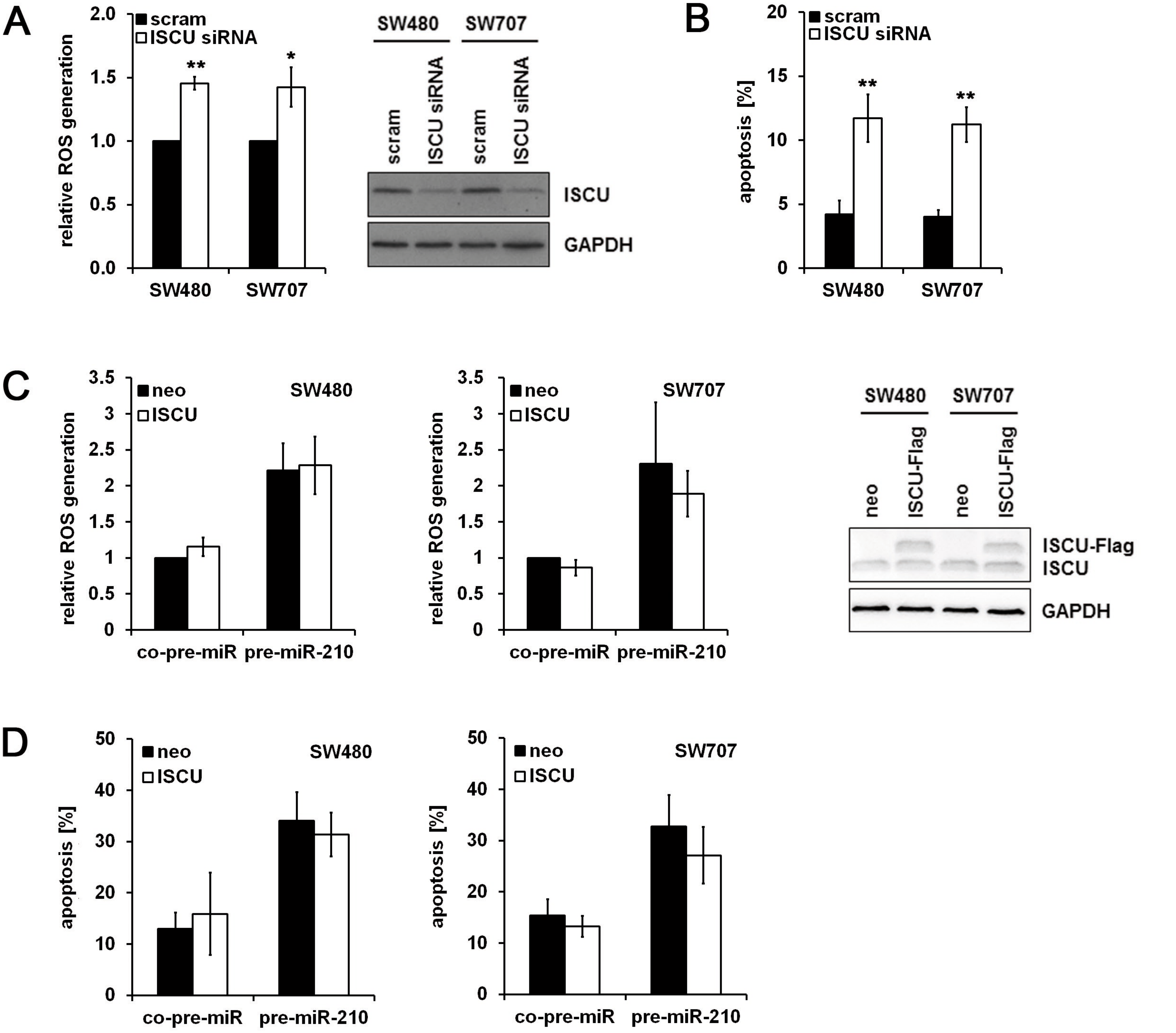

Supplemental Figure S3. A CRC cell lines were transfected with an ISCU specific siRNA and a control siRNA, respectively. ROS generation was measured 48 h after transfection (left panel; mean ± SEM; n=3; * $p < 0.05$; ** $p < 0.01$; student's t-test). Successful knockdown of ISCU was monitored by immunoblot analysis. Lysates were generated 48 h after transfection (right panel). **B** Cells were transfected as described in A. The percentage of apoptotic cells was determined 72 h post transfection by PI staining and FACS analysis. Data indicate the percentage of cells showing a sub-G1-DNA content (mean ± SEM; n=3; ** $p < 0.01$; student's t-test). **C** CRC cell lines stably overexpressing ISCU or an empty vector control (neo) were transfected with pre-miR-210 and a control miRNA, respectively. Measurement of ROS generation was performed 48 h thereafter (left panel; mean ± SEM; n=3). Successful overexpression of ISCU-Flag was monitored by immunoblot analysis. **D** Cells were transfected as described in C. The percentage of apoptotic cells was determined by PI staining and FACS analysis (mean ± SEM; n=3)
